# Calculating torque, back-EMF, inductance, and unbalanced magnetic force for a hybrid electrical vehicle by in-wheel drive application

**DOI:** 10.1038/s41598-024-63702-8

**Published:** 2024-06-05

**Authors:** Alireza Hosseinpour, Akbar Rahideh, Ahmed Abbas, Atif Iqbal, Claude Ziad El-Bayeh, Aymen Flah, Enas Ali, Ramy N. R. Ghaly

**Affiliations:** 1 Department of Electrical Engineering, Universityof Zabol, Zabol, Iran; 2 Department of Electrical and Electronics Engineering, Shiraz University of Technology, Shiraz, Iran; 3 Faculty of Electrical and Control Engineering, Gdansk University of Technology, Gdańsk, Poland; 4 Department of Electrical Engineering, Qatar University, Doha, Qatar; 5 Department of Electrical Engineering, Bayeh Institute, 55 Kfar Saleh-Hay El Arbe Street, Amchit, Mount Lebanon, Lebanon; 6 Processes, Energy, Environment, and Electrical Systems (Code: LR18ES34), National Engineering School of Gabès, University of Gabès, Gabès, Tunisia; 7 MEU Research Unit, Middle East University, Amman, Jordan; 8 College of Engineering, University of Business and Technology (UBT), 21448 Jeddah, Saudi Arabia; 9 The Private Higher School of Applied Sciences and Technologies of Gabes, University of Gabes, Gabès, Tunisia; 10 Applied Science Research Center, Applied Science Private University, Amman, 11931 Jordan; 11 ENET Centre, VSB—Technical University of Ostrava, 708 00 Ostrava, Czech Republic; 12 University Centre for Research and Development, Chandigarh University, Mohali, Punjab 140413 India; 13 Ministry of Higher Education, Mataria Technical College, Cairo, 11718 Egypt; 14 Chitkara Centre for Research and Development, Chitkara University, Chandigarh, Himachal Pradesh 174103 India

**Keywords:** Armature reaction, Auxiliary winding, Excitation coil, In-wheel drive, Transportation, Energy science and technology, Engineering

## Abstract

To use a Hybrid Excitation Synchronous Machine (HESM) in a hybrid electrical vehicle (HEV), its performance indicators such as back-EMF, inductance and unbalanced magnetic force should be computed preferably by an analytical method. First, the back-EMF is calculated by considering alternate-teeth and all-teeth non-overlapping and overlapping windings. The effects of three types of magnetization patterns including the radial, parallel and Halbach magnetizations on the back-EMF waveform have also been investigated. Then, the self-inductance of the stator and rotor windings, the mutual inductance between the stator and rotor windings, and the mutual inductance between the stator phases are computed. Next, the components of the unbalanced magnetic force (UMF) in the direction of the x and y axes and its amplitude are computed. Moreover, the effects of the magnetization patterns on those magnetic pulls are investigated. To minimize the UMFs, symmetry must be implemented in the excitation sources; therefore, first the stator winding then the permanent magnet and rotor winding are modified in such a way that the UMFs are reduced. Increasing the temperature leads to a weakening of the magnet’s residual flux density, which strongly affects the performance characteristics of the electric machine such as Back-EMF and UMF. Finally, the ratio of the permanent magnet flux to the rotor flux is determined in such a way that the average torque is maximized. In this section, the effects of three magnetization patterns will be investigated.

## Introduction

The fossil fuels not only are running out but also cause ozone layer destruction, global warming and air pollution, so using more clean energy sources is inevitable^1,2^. Therefore, hybrid and electric vehicle have been proposed^3^. Permanent magnet synchronous machines (PMSMs) have high efficiency because there is no copper loss in the rotor side. In contrast, their flux control capability is weak because PM’s flux is constant. Therefore, they aren’t appropriate choices for electric vehicle. Hybrid excitation synchronous machines (HESMs) can be utilized in hybrid electrical vehicles (HEVs), domestic applications, transportation, aircraft system and propulsion. The electric machine used as HESM has two sources for field excitation, which are permanent magnets (PM) and excitation coil (EC)^4,5^. In addition, the stator winding stimulates the armature.

The field excitations can be situated in three locations: both on the rotor, PM on the rotor and EC on the stator, and both sources on the stator^6,7^. The first case can develop the electromagnetic torque. Moreover, there is the ability of generating a constant voltage despite of the speed fluctuations. However, this topology needs brushes and retaining sleeves. If PM/EC places in the rotor/stator, the machine does not require any brush and produces electromagnetic torque, but the presence of a retaining sleeve is inevitable, and it is one of its weaknesses. The application of this HESM is at high-speed applications. In the third structure the stator includes both sources, so the brushes and the retaining sleeve are eliminated, but the mutual torque is not produced, because the rotor is inactive. This type of machine can be used at very high speeds because it has no excitation in the rotary part. A HESM should have a constant voltage despite of the speed fluctuations, the voltage can be controlled by sensor-less drive method^8,9^. However, the purpose of this manuscript is to determine the appropriate structure for it. Therefore, it is better to place both field excitation sources in the rotor so that the flux of the main source (PM) can be controlled by using an auxiliary source (EC). Its efficiency is less than PMSM because rotor winding losses have been added. In contrast, its current is low, so it doesn’t significantly effect. Further, EC is caused the machine speed is limited. However, transportation is a low and medium speed application, so it is an appropriate option for it. Consequently, it consists of both PMSM’s advantages and flux control capability. This type of machine can be utilized in variable speed applications in transportation systems such as in-wheel drive^10,11^. Three distinct variants have been proposed for this structure: the spoke type, the buried, and the surface-mounted PM machines. The first one is the spoke type machine^12^ in which an analytical method (AM), based on the subdomain method, is presented for calculating the open circuit and the armature reaction magnetic field. The proposed model takes the tooth of the rotor and the stator slots, and the shape of the poles into account. This method involves solving Poisson’s and Laplace’s equations in the semi- closed slots of the rotor and stator, the air gap, and PMs in the rotor slots. Given that, the spoke machine can accommodate a large number of poles, which is suitable for low-speed applications such as wind turbines^13^. The spoke machine can also use an auxiliary magnet instead of the excitation coil^14^. In this paper, similar to the previous one, at first, the magnetic field in all parts of HESM is determined by two-dimensional analytical method. Then, the electromagnetic torque and the back-EMF are computed. In addition, changes in the arrangement of the magnets and the materials have also been investigated^15–19^. The topics related to the auxiliary magnets, such as magnetization patterns and the geometric structure, have not been discussed in this study, but they can be considered in the future research.

The second topology that uses both PM and EC excitations in the rotor is the buried machine^17,18^. The armature reaction is considered. However, the analysis performed is based on the finite element method (FEM). Inductances in direct and quadrature axes, the air gap flux density, the efficiency, and the terminal voltage are calculated. This type of HESM is used as a generator, for example in a diesel generator, in the islanding mode of operation.

**Figure 1:**
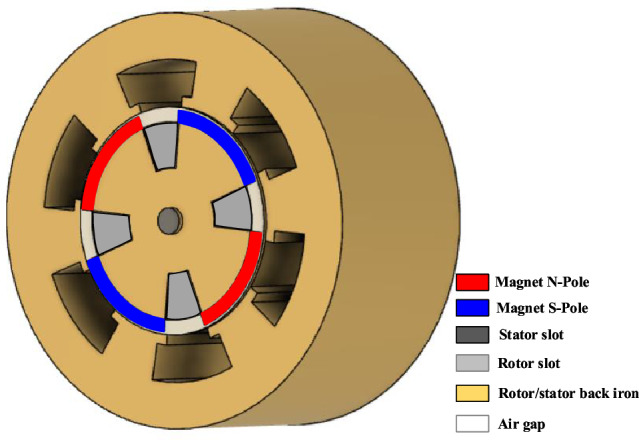
SHESM with two excitation sources on the rotor.

The last structure of HESM having two excitation fields in the rotor is the surface mounted permanent magnet machine^16^, which was presented in Fig. 1. In this machine, the auxiliary winding flux passes through the magnet; therefore, it is a series HESM (SHESM). In^19^ no-load analysis has been performed and the distribution of the magnetic field in all parts of the machine has been calculated by AM with desirable accuracy.

The cogging torque and the back-EMF are determined. In subsequent studies, the eddy current losses in no-load condition and the effect of the fragmentation of the magnets have been investigated^20^. Neither the armature reaction nor the study on different magnetization patterns and different stator windings are considered. Therefore, a comprehensive model that takes into account the armature reaction, different magnetization patterns and different winding layouts is necessary to compute all quantities of the HESM. This machine is analyzed as statically^21^. As well as the magnetic field is calculated. Moreover, a HESM is studied by a numerical method^22^. The torque, unbalance magnetic force, self- and mutual inductance of stator winding have been computed. In this paper, dynamic analysis is performed.

In the present manuscript, based on the analysis done in^21^, first, the magnetic field is obtained in all parts of the HESM including the airgap, the rotor and stator slots, the slot-openings and the PMs. Second, by means of the obtained magnetic field, the back-EMF is determined by considering alternate-teeth and all-teeth non-overlapping windings and overlapping one. The effects of the radial, parallel and Halbach magnetization patterns on the back-EMF are also investigated. Because the HESM in an HEV should be fed by a low harmonic voltage, an optimal topology of pole number, slots, magnetization pattern and stator winding to obtain a sinusoidal voltage is provided. Third, the self-inductance of the rotor and the stator windings, the mutual inductance between the stator phases, and the mutual inductance between the stator and rotor windings are calculated. Forth, the unbalance magnetic forces (UMF) are computed. In addition, different magnetization patterns and non-overlapping and overlapping stator winding are taken into account. Then, some methods are proposed to reduce these magnetic pulls. It is noted that not only in HEVs but also in most applications, an electric machine with low UMF is preferred. Next, the temperature effect on Back-EMF and UMF has been investigated. After that, in-wheel drive requirements such as efficiency, motor power and rotational speed will be scrutinized. Finally, average torque in terms of magnetization index for radial, parallel and Halbach magnetization patterns have been illustrated. All results have been validated by a numerical method. The proposed machine not only has not been dynamically analyzed by 2-D method but also the winding, magnetization and temperature impacts have not been previously considered.

## Methodology

### Magnetic flux density

The integral constants are achieved by solving the simultaneous equation (65) in^21^. Having the computed integral constants, the radial and tangential components of the magnetic flux density based on the magnetic vector potential in all regions can be found:

1 $${B}_{r}^{sls,j}\left(r,\theta \right)=-\sum_{{v}_{s}=1}^{{V}_{s}}\frac{\pi {v}_{s}}{{\delta }_{s}}\left\{\frac{{b}_{{v}_{s}}^{sls,j}}{{R}_{so}}\left[{\left(\frac{{R}_{so}}{{R}_{sl}}\right)}^{\frac{\pi {v}_{s}}{{\delta }_{s}}+1}{\left(\frac{r}{{R}_{sl}}\right)}^{\frac{\pi {v}_{s}}{{\delta }_{s}}-1}+{\left(\frac{{R}_{so}}{r}\right)}^{\frac{\pi {v}_{s}}{{\delta }_{s}}+1}\right]\right.+\left.\frac{{\mu }_{0}{J}_{{v}_{s}}^{i}}{{\left(\frac{\pi {v}_{s}}{{\delta }_{s}}\right)}^{2}-4}\left[r-\frac{2{R}_{sl}}{\frac{\pi {v}_{s}}{{\delta }_{s}}}{\left(\frac{r}{{R}_{sl}}\right)}^{\frac{\pi {v}_{s}}{{\delta }_{s}}-1}\right]\right\}\times sin(\frac{\pi {v}_{s}}{{\delta }_{s}}(\theta -{\theta }_{j}+\frac{{\delta }_{s}}{2}))$$

2 $${B}_{\theta }^{sls,j}\left(r,\theta \right)=\frac{-{\mu }_{0}{J}_{0}^{i}}{2}\left[\frac{{R}_{sl}^{2}}{r}-r\right]-\sum_{{v}_{s}=1}^{{V}_{s}}\frac{\pi {v}_{s}}{{\delta }_{s}}\left\{\frac{{b}_{{v}_{s}}^{sls,j}}{{R}_{so}}\left[{\left(\frac{{R}_{so}}{{R}_{sl}}\right)}^{\frac{\pi {v}_{s}}{{\delta }_{s}}+1}{\left(\frac{r}{{R}_{sl}}\right)}^{\frac{\pi {v}_{s}}{{\delta }_{s}}-1}-{\left(\frac{{R}_{so}}{r}\right)}^{\frac{\pi {v}_{s}}{{\delta }_{s}}+1}\right]+\frac{2{\mu }_{0}{J}_{{v}_{s}}^{i}}{{\left(\frac{\pi {v}_{s}}{{\delta }_{s}}\right)}^{2}-4}\left[r-{R}_{sl}{\left(\frac{r}{{R}_{sl}}\right)}^{\frac{\pi {v}_{s}}{{\delta }_{s}}-1}\right]\right\}\times cos(\frac{\pi {v}_{s}}{{\delta }_{s}}(\theta -{\theta }_{j}+\frac{{\delta }_{s}}{2})$$

3 $${B}_{r}^{so,j}\left(r,\theta \right)=-\sum_{u=1}^{U}\frac{\pi u}{\beta }\left\{\frac{{a}_{u}^{so,j}}{{R}_{so}}{\left(\frac{r}{{R}_{so}}\right)}^{\frac{\pi u}{\beta }-1}+\frac{{b}_{u}^{so,j}}{{R}_{s}}{\left(\frac{{R}_{s}}{r}\right)}^{\frac{\pi u}{\beta }+1}\right\}\times \text{sin}(\frac{\pi u}{\beta }(\theta -{\theta }_{j}+\frac{\beta }{2}))$$

4 $${B}_{r}^{so,j}\left(r,\theta \right)=\frac{{b}_{0}^{so,j}}{r}-\sum_{u=1}^{U}\frac{\pi u}{\beta }\left\{\frac{{a}_{u}^{so,j}}{{R}_{so}}{\left(\frac{r}{{R}_{so}}\right)}^{\frac{\pi u}{\beta }-1}-\frac{{b}_{u}^{so,j}}{{R}_{s}}{\left(\frac{{R}_{s}}{r}\right)}^{\frac{\pi u}{\beta }+1}\right\}\times \text{cos}(\frac{\pi u}{\beta }(\theta -{\theta }_{j}+\frac{\beta }{2}))$$

5 $${B}_{r}^{m}\left(r,\theta \right)=\sum_{n=1}^{N}n\left\{-\left[\frac{{a}_{n}^{m}}{{R}_{m}}{\left(\frac{r}{{R}_{m}}\right)}^{n-1}+\frac{{b}_{n}^{m}}{{R}_{r}}{\left(\frac{{R}_{r}}{r}\right)}^{n+1}-{k}_{n}\right]\times \mathit{sin}\left(n\alpha \right)\mathit{sin}\left(n\theta \right)\right.\left.+\left[\frac{{c}_{n}^{m}}{{R}_{m}}{\left(\frac{r}{{R}_{m}}\right)}^{n-1}+\frac{{d}_{n}^{m}}{{R}_{r}}{\left(\frac{{R}_{r}}{r}\right)}^{n+1}+{k}_{n}\right]\times cos(n\alpha )cos(n\theta )\right\}$$

6 $$\begin{gathered} B_{\theta }^{m} \left( {r,\theta } \right) = \frac{{b_{0}^{m} }}{r} - \mathop \sum \limits_{n = 1}^{N} n\left\{ {\left[ {\frac{{a_{n}^{m} }}{{R_{m} }}\left( {\frac{r}{{R_{m} }}} \right)^{n - 1} - \frac{{b_{n}^{m} }}{{R_{r} }}\left( {\frac{{R_{r} }}{r}} \right)^{n + 1} - \frac{1}{n}\frac{{dk_{n} r}}{dr}} \right] \times \sin \left( {n\alpha } \right)\cos \left( {n\theta } \right)} \right. \hfill \\ \left. { + \left[ {\frac{{c_{n}^{m} }}{{R_{m} }}\left( {\frac{r}{{R_{m} }}} \right)^{n - 1} - \frac{{d_{n}^{m} }}{{R_{r} }}\left( {\frac{{R_{r} }}{r}} \right)^{n + 1} + \frac{1}{n}\frac{{dk_{n} r}}{dr}} \right]{\text{cos}}\left( {n\alpha } \right){\text{sin}}\left( {n\theta } \right)} \right\} \hfill \\ \end{gathered}$$

7 $${B}_{r}^{slr,i}\left(r,\theta \right)=-\sum_{{v}_{s}=1}^{{V}_{s}}\frac{\pi {v}_{r}}{{\delta }_{r}}\left\{\frac{{a}_{{v}_{r}}^{slr,i}}{{R}_{r}}\left[{\left(\frac{r}{{R}_{r}}\right)}^{\frac{\pi {v}_{r}}{{\delta }_{r}}-1}+{{\left(\frac{{R}_{1}}{{R}_{r}}\right)}^{\frac{\pi {v}_{r}}{{\delta }_{r}}-1}\left(\frac{{R}_{1}}{r}\right)}^{\frac{\pi {v}_{r}}{{\delta }_{r}}+1}\right]\right\}\times \text{sin}(\frac{\pi {v}_{r}}{{\delta }_{r}}(\theta -{\theta }_{i}+\frac{{\delta }_{r}}{2}))$$

8 $${B}_{\theta }^{slr,i}\left(r,\theta \right)=\frac{-{\mu }_{0}{J}_{0}^{i}}{2}\left[\frac{{R}_{1}^{2}}{r}-r\right]-\sum_{{v}_{r}=1}^{{V}_{r}}\frac{\pi {v}_{r}}{{\delta }_{r}}\left\{\frac{{a}_{{v}_{r}}^{slr,i}}{{R}_{r}}\times \left[{\left(\frac{r}{{R}_{r}}\right)}^{\frac{\pi {v}_{r}}{{\delta }_{r}}-1}-{\left(\frac{{R}_{r}}{{R}_{r}}\right)}^{\frac{\pi {v}_{r}}{{\delta }_{r}}-1}{\left(\frac{{R}_{1}}{r}\right)}^{\frac{\pi {v}_{r}}{{\delta }_{r}}+1}\right]\right\}\times \text{cos}(\frac{\pi {v}_{r}}{{\delta }_{r}}(\theta -{\theta }_{i}+\frac{{\delta }_{r}}{2}))$$

9 $${B}_{r}^{a}\left(r,\theta \right)=-\sum_{n=1}^{N}n\left\{\left[\frac{{a}_{n}^{a}}{{R}_{s}}{\left(\frac{r}{{R}_{s}}\right)}^{n-1}+\frac{{b}_{n}^{a}}{{R}_{m}}{\left(\frac{{R}_{m}}{r}\right)}^{n+1}\right]\times \text{sin}\left(n\theta \right)-\left[\frac{{c}_{n}^{a}}{{R}_{s}}{\left(\frac{r}{{R}_{s}}\right)}^{n-1}+\frac{{d}_{n}^{a}}{{R}_{m}}{\left(\frac{{R}_{m}}{r}\right)}^{n+1}\right]\times \text{cos}(n\theta )\right\}$$

10 $${B}_{\theta }^{a}\left(r,\theta \right)=-\sum_{n=1}^{N}n\left\{\left[\frac{{a}_{n}^{a}}{{R}_{s}}{\left(\frac{r}{{R}_{s}}\right)}^{n-1}-\frac{{b}_{n}^{a}}{{R}_{m}}{\left(\frac{{R}_{m}}{r}\right)}^{n+1}\right]\times \text{cos}\left(n\theta \right)+\left[\frac{{c}_{n}^{a}}{{R}_{s}}{\left(\frac{r}{{R}_{s}}\right)}^{n-1}-\frac{{d}_{n}^{a}}{{R}_{m}}{\left(\frac{{R}_{m}}{r}\right)}^{n+1}\right]\times \text{sin}(n\theta )\right\}$$

*Sls*, so, *m*, *slr* and *a* are slot of stator, slot-opening, magnet, slot of rotor and airgap, respectively. Other quantities such as the back-EMF, the inductance and the unbalance magnetic force are determined in the following sub-sections.

### Back-EMF

**Figure 2:**
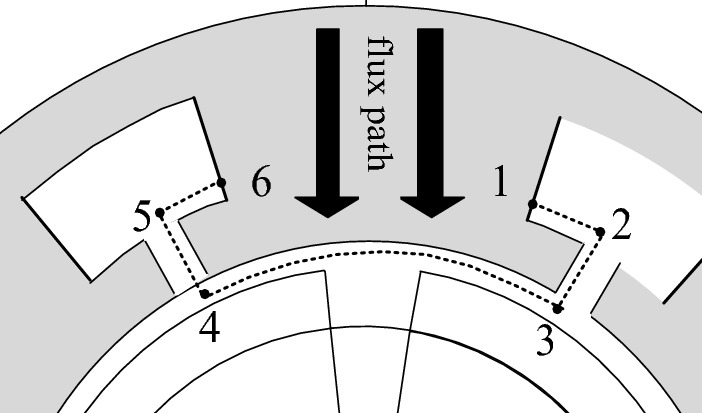
Flux path in teeth.

The path of the flux passing through a stator tooth is shown in Fig. 2. It can be computed with Gauss’s law.

11 $$\oint B.ds=0{\to \varphi }_{16}={\varphi }_{12}+{\varphi }_{23}+{\varphi }_{34}+{\varphi }_{45}+{\varphi }_{56}=\underset{{s}_{12}}{{\int }}B.ds+\underset{{s}_{23}}{{\int }}B.ds+\underset{{s}_{34}}{{\int }}B.ds+\underset{{s}_{45}}{{\int }}B.ds+\underset{{s}_{56}}{{\int }}B.ds$$

Using constant coefficients in the analytical model of the machine, the flux of one turn of *j*th winding, which is 16 wrapped on the *j*th tooth in the non-overlapping winding, is obtained from the following equation:

12 $${\varphi }_{j}\left(\alpha \right)=L\sum_{{v}_{s}=1}^{{V}_{s}}\left[{\left(\frac{{R}_{so}}{{R}_{sl}}\right)}^{\frac{2\pi {v}_{s}}{{\delta }_{s}}}+1\right]\times \left\{{b}_{{v}_{s}}^{sls,j}\left[\text{cos}\left(\frac{\pi {v}_{s}}{{\delta }_{s}}\left(\frac{{\delta }_{s}+\beta }{2}\right)\right)-{(-1)}^{{v}_{s}}\right]-{b}_{{v}_{s}}^{sls,j+1}\left[\text{cos}\left(\frac{\pi {v}_{s}}{{\delta }_{s}}\left(\frac{{\delta }_{s}-\beta }{2}\right)\right)-1\right]\right\}+\text{L}\sum_{u=1}^{U}{[(a}_{u}^{so,j+1}-{\left(-1\right)}^{u}{a}_{u}^{so,j})-{(b}_{u}^{so,j+1}-{\left(-1\right)}^{u}{b}_{u}^{so,j})]\left[{\left(\frac{{R}_{s}}{{R}_{so}}\right)}^{\frac{\pi u}{\beta }}-1\right]-2L\sum_{n=1}^{N}\left\{\left[{a}_{n}^{a}+{b}_{n}^{a}{\left(\frac{{R}_{m}}{{R}_{s}}\right)}^{n}\right]\text{sin}\left(n{\delta }_{j+1}\right)-\left[{c}_{n}^{a}+{d}_{n}^{a}{\left(\frac{{R}_{m}}{{R}_{s}}\right)}^{n}\right]\text{cos}(n{\delta }_{j+1})\right\}\text{sin}(\frac{n{\theta }_{t}}{2})$$

In the case of all-teeth wound we have *j*=*1,2,…,Qj*. In contrast, *j*=*1,3,5,…,Q*_*j*_*-1* for the case of alternate-teeth wound. Moreover, *θ*_*t*_=*2π/Q*_*j*_*-β* is the span angle of each tooth and *δ*_*j*_ = *2π(j − 1)/Q*_*j*_ is the angle of *j*th stator slot center. In addition, the flux of one turn of *j*th winding, which is wrapped on the *j* and *j*+*s* teeth in the non-overlapping winding, is obtained from the following equation.

13 $${\varphi }_{j}\left(\alpha \right)=L\sum_{{v}_{s}=1}^{{V}_{s}}\left[{\left(\frac{{R}_{so}}{{R}_{sl}}\right)}^{\frac{2\pi {v}_{s}}{{\delta }_{s}}}+1\right]\times \left\{{b}_{{v}_{s}}^{sls,j}\left[\text{cos}\left(\frac{\pi {v}_{s}}{{\delta }_{s}}\left(\frac{{\delta }_{s}+\beta }{2}\right)\right)-{(-1)}^{{v}_{s}}\right]-{b}_{{v}_{s}}^{sls,j+1}\left[\text{cos}\left(\frac{\pi {v}_{s}}{{\delta }_{s}}\left(\frac{{\delta }_{s}-\beta }{2}\right)\right)-1\right]\right\}+\text{L}\sum_{u=1}^{U}{[(a}_{u}^{so,j+1}-{\left(-1\right)}^{u}{a}_{u}^{so,j})-{(b}_{u}^{so,j+1}-{\left(-1\right)}^{u}{b}_{u}^{so,j})]\left[{\left(\frac{{R}_{s}}{{R}_{so}}\right)}^{\frac{\pi u}{\beta }}-1\right]-2L\sum_{n=1}^{N}\left\{\left[{a}_{n}^{a}+{b}_{n}^{a}{\left(\frac{{R}_{m}}{{R}_{s}}\right)}^{n}\right]\text{sin}\left(n{\delta }_{j+(s+1)/2}\right)-\left[{c}_{n}^{a}+{d}_{n}^{a}{\left(\frac{{R}_{m}}{{R}_{s}}\right)}^{n}\right]\text{cos}(n{\delta }_{j+(s+1)/2})\right\}\text{sin}(\frac{n\pi s}{Q}-\frac{n\beta }{2})$$

The induced voltage in the *j*th windings can be achieved using Faraday’s law of induction.

14 $${E}_{j}=-{N}_{t}\frac{d{\varphi }_{j}}{d\alpha }$$

In this method, the magnetic flux density in the air gap, the stator slot-opening and the slot is utilized to calculate the induced voltage. Both overlapping and non-overlapping windings can be considered.

### Inductance

To compute the self and mutual inductances of the stator phases, the flux density caused by the armature current should solely be considered. The flux linkage of *j*th winding caused by the *j’* phase flowing in the non-overlapping windings is calculated from the following equation.

15 $${\lambda }_{j,j{\prime}}\left(\alpha \right)=L{N}_{t}\sum_{{v}_{s}=1}^{{V}_{s}}\left[{\left(\frac{{R}_{so}}{{R}_{sl}}\right)}^{\frac{2\pi {v}_{s}}{{\delta }_{s}}}+1\right]\times \left\{{b}_{{v}_{s}}^{sls,j,{j}{\prime}}\left[\text{cos}\left(\frac{\pi {v}_{s}}{{\delta }_{s}}\left(\frac{{\delta }_{s}+\beta }{2}\right)\right)-{\left(-1\right)}^{{v}_{s}}\right]-{b}_{{v}_{s}}^{sls,j+1,j{\prime}}\left[\text{cos}\left(\frac{\pi {v}_{s}}{{\delta }_{s}}\left(\frac{{\delta }_{s}-\beta }{2}\right)\right)-1\right]\right\}+\left({(b}_{0}^{so,j+1,j{\prime}}-{(b}_{0}^{so,j,j{\prime}}\right)\text{ln}\left(\frac{{R}_{s}}{{R}_{so}}\right)+L{N}_{t}\sum_{{v}_{s}}^{{V}_{s}}\frac{{\mu }_{0}}{{\left(\frac{\pi {v}_{s}}{{\delta }_{s}}\right)}^{2}-4}\left[{R}_{so}^{2}+\frac{2{R}_{sl}^{2}}{\frac{\pi {v}_{s}}{{\delta }_{s}}}{\left(\frac{{R}_{so}}{{R}_{sl}}\right)}^{\frac{\pi {v}_{s}}{{\delta }_{s}}}\right]\left\{{J}_{{v}_{s}}^{j,j{\prime}}\left[\text{cos}\left(\frac{\pi {v}_{s}}{{\delta }_{s}}\left(\frac{{\delta }_{s}+\beta }{2}\right)\right)-{(-1)}^{{v}_{s}}\right]-{J}_{{v}_{s}}^{j+1,j{\prime}}\left[\text{cos}\left(\frac{\pi {v}_{s}}{{\delta }_{s}}\left(\frac{{\delta }_{s}-\beta }{2}\right)\right)-1\right]\right\}+\text{L}{N}_{t}\sum_{u=1}^{U}{[(a}_{u}^{so,j+1,j{\prime}}-{\left(-1\right)}^{u}{a}_{u}^{so,j,j{\prime}})-{(b}_{u}^{so,j+1,j{\prime}}-{\left(-1\right)}^{u}{b}_{u}^{so,j,j{\prime}})]\left[{\left(\frac{{R}_{s}}{{R}_{so}}\right)}^{\frac{\pi u}{\beta }}-1\right]-2L{N}_{t}\sum_{n=1}^{N}\left\{\left[{a}_{n}^{a,j{\prime}}+{b}_{n}^{a,j{\prime}}{\left(\frac{{R}_{m}}{{R}_{s}}\right)}^{n}\right]\text{sin}\left(n{\delta }_{j+1}\right)-\left[{c}_{n}^{a,j{\prime}}+{d}_{n}^{a,j{\prime}}{\left(\frac{{R}_{m}}{{R}_{s}}\right)}^{n}\right]\text{cos}(n{\delta }_{j+1})\right\}\text{sin}(\frac{n{\theta }_{t}}{2})$$

For *j* = *1,2,…,Q*_*j*_ and *j’* = *1,2,…,Q*_*j*_; and ${J}_{{v}_{s}}^{j,j{\prime}}$ is the *v*_*s*_ component of the current density in slot *j* when all phases currents are zero except for *j’*.

In addition, the flux linkage of *j*th windings (between *j*th and (*j*+*s*)th teeth) caused by the *j’* phase flowing in the non-overlapping windings is computed from the following equation.

16 $${\lambda }_{j,j{\prime}}\left(\alpha \right)=L{N}_{t}\sum_{{v}_{s}=1}^{{V}_{s}}\left[{\left(\frac{{R}_{so}}{{R}_{sl}}\right)}^{\frac{2\pi {v}_{s}}{{\delta }_{s}}}+1\right]\times \left\{{b}_{{v}_{s}}^{sls,j,j{\prime}}\left[\text{cos}\left(\frac{\pi {v}_{s}}{{\delta }_{s}}\left(\frac{{\delta }_{s}+\beta }{2}\right)\right)-{(-1)}^{{v}_{s}}\right]-{b}_{{v}_{s}}^{sls,j+1,j{\prime}}\left[\text{cos}\left(\frac{\pi {v}_{s}}{{\delta }_{s}}\left(\frac{{\delta }_{s}-\beta }{2}\right)\right)-1\right]\right\}+\left({(b}_{0}^{so,j+1,j{\prime}}-{(b}_{0}^{so,j,j{\prime}}\right)\text{ln}\left(\frac{{R}_{s}}{{R}_{so}}\right)+\text{L}{N}_{t}\sum_{u=1}^{U}{[(a}_{u}^{so,j+1,j{\prime}}-{\left(-1\right)}^{u}{a}_{u}^{so,j,j{\prime}})-{(b}_{u}^{so,j+1,j{\prime}}-{\left(-1\right)}^{u}{b}_{u}^{so,j,j{\prime}})]\left[{\left(\frac{{R}_{s}}{{R}_{so}}\right)}^{\frac{\pi u}{\beta }}-1\right]-2L{N}_{t}\sum_{n=1}^{N}\left\{\left[{a}_{n}^{a,j{\prime}}+{b}_{n}^{a,j{\prime}}{\left(\frac{{R}_{m}}{{R}_{s}}\right)}^{n}\right]\text{sin}\left(n{\delta }_{j+1}\right)-\left[{c}_{n}^{a,j{\prime}}+{d}_{n}^{a,j{\prime}}{\left(\frac{{R}_{m}}{{R}_{s}}\right)}^{n}\right]\text{cos}(n{\delta }_{j+1})\right\}\text{sin}(\frac{n{\theta }_{t}}{2})$$

In the case of all-teeth wound we have *j*=*1,2,…,Qj*. In contrast, *j*=*1,3,5,…,Q*_*j*_*-1* for the case of alternate-teeth wound. If there are several series windings in *j*th windings phase, their fluxes should be added together. Finally, the *k*th phase inductance due to the *k’* phase current is calculated from the following equation.

17 $${L}_{k,k{\prime}}=\sum_{j\epsilon k,j{\prime}\epsilon k{\prime}}\frac{{\lambda }_{j,j{\prime}}}{{i}_{j{\prime}}}$$

To achieve the self-inductance, it is required to excite phase k instead of k’. Next, the flux linkage caused by this current is computed. Finally, the self-inductance is determined by the following equation.

18 $${L}_{k,k}=\sum_{j\epsilon k,j{\prime}\epsilon k}\frac{{\lambda }_{j,j{\prime}}}{{i}_{j{\prime}}}$$

The mutual inductance between the stator and the rotor windings can be calculated by (17), in this regard, the rotor winding is excited and the other field excitation sources are inactive. The constant coefficients in equation (65) in^21^ are determined according to the rotor excitation.

To compute the self-inductance of the rotor winding, the flux linkage of the rotor winding should be determined. It is calculated by (19)

19 $${\lambda }_{j,j}={N}_{r}L\left\{\frac{{b}_{0}^{m}{\delta }_{r}}{{R}_{r}}-2\sum_{n=1}^{N}\left[\frac{{a}_{n}^{m}}{{R}_{m}}{\left(\frac{{R}_{r}}{{R}_{m}}\right)}^{n-1}-\frac{{b}_{n}^{m}}{{R}_{r}}-\frac{1}{n}\frac{d{k}_{n}r}{dr}\right]\left[\text{sin}\left(n\alpha \right)\text{sin}\left(n\left(\frac{\pi }{{Q}_{r}}-\frac{{\delta }_{r}}{2}\right)\right)\text{cos}\left(n\left(\frac{{\theta }_{j+1}+{\theta }_{j}}{2}\right)\right)\right]+\left[\frac{{c}_{n}^{m}}{{R}_{m}}{\left(\frac{{R}_{r}}{{R}_{m}}\right)}^{n-1}-\frac{{d}_{n}^{m}}{{R}_{r}}+\frac{1}{n}\frac{d{k}_{n}r}{dr}\right]\left[\text{cos}\left(n\alpha \right)\text{sin}\left(n\left(\frac{\pi }{{Q}_{r}}-\frac{{\delta }_{r}}{2}\right)\right)\text{sin}\left(n\left(\frac{{\theta }_{j+1}+{\theta }_{j}}{2}\right)\right)\right]-\sum_{j=1}^{{N}_{r}}\frac{{\pi v}_{r}}{{\delta }_{r}} \frac{{a}_{{v}_{r}}^{slr,j}}{{\delta }_{r}} \left[1-{\left(\frac{{R}_{1}}{{R}_{r}}\right)}^{\frac{{\pi v}_{r}}{{\delta }_{r}}}\right]\left[-{\pi v}_{r}+\left({\pi v}_{r}-{\delta }_{r}\right){\left(\frac{{R}_{1}}{{R}_{r}}\right)}^{\frac{{\pi v}_{r}}{{\delta }_{r}}+1}\right]sin\left(\frac{{\pi v}_{r}}{{\delta }_{r}}\left({\theta }_{j+1}-{\theta }_{j}\right)\right)\right\}$$

For *j*=*1,2,…,Q*_*j*_ and the *v*_*r*_ is component of the current density in slot *j*.

The self-inductance of the rotor winding is determined by the following equation.

20 $${L}_{RR}=\sum_{j\epsilon k}\frac{{\lambda }_{j,j}}{{i}_{j}}$$

The flux density in the air gap, the magnet, the rotor slot, the slot opening, and the stator slot are used to determine the inductances.

### Unbalanced magnetic force

Maxwell stress tensor is used to compute the radial and tangential forces which are exerted into the stator surface. These forces are calculated based on the magnetic fields in the airgap as following.

21 $${f}_{r}=\frac{1}{2{\mu }_{0}}\left({{B}_{r}}^{2}-{{B}_{\theta }}^{2}\right)$$

22 $${{f}_{\theta }=\frac{1}{{\mu }_{0}}B}_{r}{B}_{\theta }$$

These equations can be transformed into the Cartesian system.

23 $${f}_{x}={f}_{r}\text{cos}\left(\theta \right)-{f}_{\theta }\text{sin}(\theta )$$

24 $${f}_{y}={f}_{r}\text{sin}\left(\theta \right)+{f}_{\theta }\text{cos}(\theta )$$

Then, UMF is determined with the integration of these forces in the middle of the air gap.

25 $${F}_{x}\left(t\right)=\underset{-L/2}{\overset{L/2}{\int }}\underset{-\pi }{\overset{\pi }{\int }}{f}_{x}rd\theta dz=L\underset{-\pi }{\overset{\pi }{\int }}{f}_{x}rd\theta$$

26 $${F}_{y}\left(t\right)=\underset{-L/2}{\overset{L/2}{\int }}\underset{-\pi }{\overset{\pi }{\int }}{f}_{y}rd\theta dz=L\underset{-\pi }{\overset{\pi }{\int }}{f}_{y}rd\theta$$

27 $${F}_{r}=\sqrt{{F}_{x}^{2}+{F}_{y}^{2}}$$

The magnitude of the unbalance magnetic force and its components are computed due to only the air gap magnetic flux density.

### Temperature effect

If the temperature in a magnet increase, its residual flux density decreases. If the temperature changes in the range of 20–150 °C degrees and the PM type is selected to be Nd–Fe–B^23^, then the residual flux density varies based on the following expression.

28 $${B}_{r}={B}_{r20}\left[1+\frac{{\alpha }_{B}}{100}\left({\theta }_{PM}-20\right)\right]$$

where $${\alpha }_{B}$$ is 0.105.

Therefore, temperature directly affects the Back-EMF and UMF, which will be investigated.

### Electromagnetic Torque

The instantaneous torque (*T*_*inst*_) of a SHESM is calculated based on Maxwell stress tensor according to equation (29).

29 $${T}_{inst}=\frac{L}{{u}_{0}}\underset{-\pi }{\overset{\pi }{\int }}\left({B}_{r,PM}^{a}+{B}_{r,EC}^{a}+{B}_{r,AR}^{a}\right)\left({B}_{\theta ,PM}^{a}+{B}_{\theta ,EC}^{a}+{B}_{\theta ,AR}^{a}\right)\left|\begin{array}{c} \\ \begin{array}{c} \\ \\ r={R}_{c}\end{array}\end{array}\right.{R}_{c}^{2}d\theta$$

where ${B}_{r,PM}^{a}, {B}_{r,EC}^{a}, {B}_{r,AR}^{a}, {B}_{\theta ,PM}^{a}, {B}_{\theta ,EC}^{a} \text{and} {B}_{\theta ,AR}^{a}$ are the radial and tangential components of the magnetic flux density due to PMs, EC and AR in the air-gap, respectively.

Equation (29) calculates the total torque. Cogging (cog), Reluctance (re), and mutual (mu) torque waveforms can be obtained separately from equations (30) to (32).

30 $${T}_{cog}=\frac{L}{{u}_{0}}\underset{-\pi }{\overset{\pi }{\int }}\left({B}_{r,PM}^{a}+{B}_{r,EC}^{a}\right)\left({B}_{\theta ,PM}^{a}+{B}_{\theta ,EC}^{a}\right)\left|\begin{array}{c} \\ \begin{array}{c} \\ \\ r={R}_{c}\end{array}\end{array}\right.{R}_{c}^{2}d\theta$$

31 $${T}_{re}=\frac{L}{{u}_{0}}\underset{-\pi }{\overset{\pi }{\int }}\left({B}_{r,AR}^{a}\right)\left({B}_{\theta ,AR}^{a}\right)\left|\begin{array}{c} \\ \begin{array}{c} \\ \\ r={R}_{c}\end{array}\end{array}\right.{R}_{c}^{2}d\theta$$

32 $${T}_{mu}=\frac{L}{{u}_{0}}\underset{-\pi }{\overset{\pi }{\int }}\left({B}_{r,AR}^{a}\right)\left({B}_{\theta ,PM}^{a}+{B}_{\theta ,EC}^{a}\right)+\left({B}_{\theta ,AR}^{a}\right)\left({B}_{r,PM}^{a}+{B}_{r,EC}^{a}\right)\left|\begin{array}{c} \\ \begin{array}{c} \\ \\ r={R}_{c}\end{array}\end{array}\right.{R}_{c}^{2}d\theta$$

## Case Study

### Computation of Indicators

The back-EMF, all inductances and the UMF are calculated for a series HESM whose characteristics are presented in Table 1. In order to compute those quantities, the radial, parallel and Halbach magnetization patterns are taken into account.

**Table 1:** Parameters of the SHESM with the alternate-teeth winding.

Parameter	Values	Parameter	Values
p	1	δ_s_	0.6283 rad
Q_s_	6	β	0.4398 rad
Q_r_	2	δ_r_	0.2356 rad
R_o_	105 mm	α_p_	0.85
R_sl_	81.5 mm	B_rem_	1
R_so_	62.9 mm	μ_rm_	1.05
R_s_	57.5 mm	N, U, V_s_, V_r_	100
R_m_	56 mm	I_r_	10 A
R_r_	50 mm	I_m_	14.14 A
R_1_	26.5 mm	k_f_	0.6

**Figure 3:**
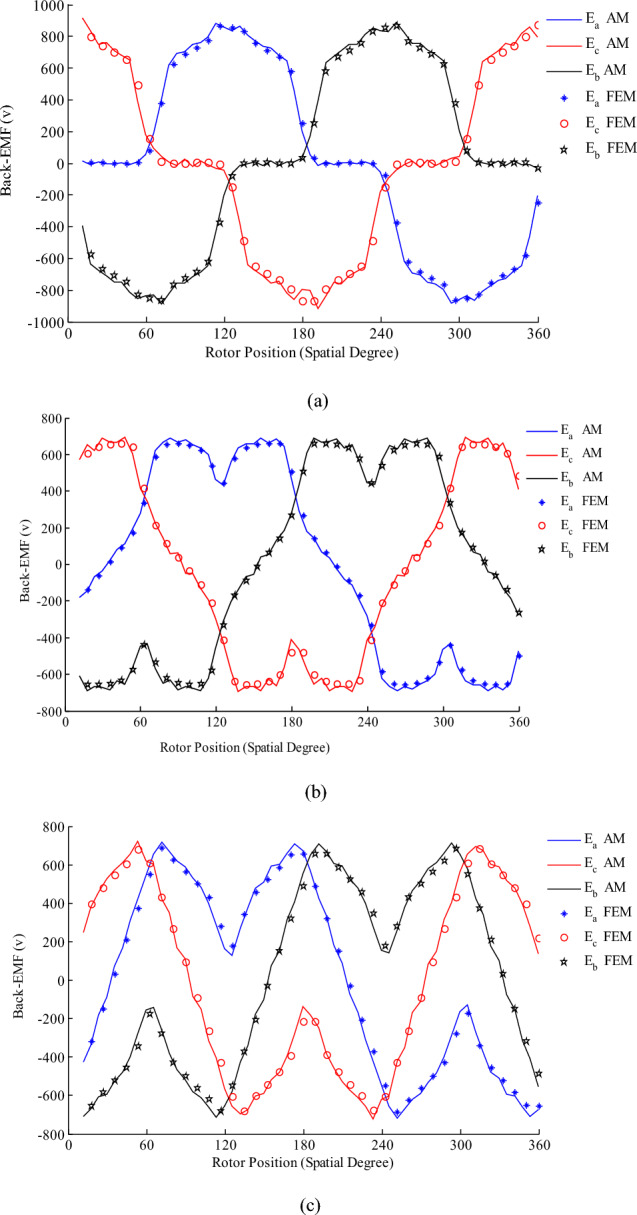
Back-EMF for alternate-teeth non-overlapping winding (**a**) radial magnetization (**b**) parallel magnetization (**c**) Halbach magnetization.

**Figure 4:**
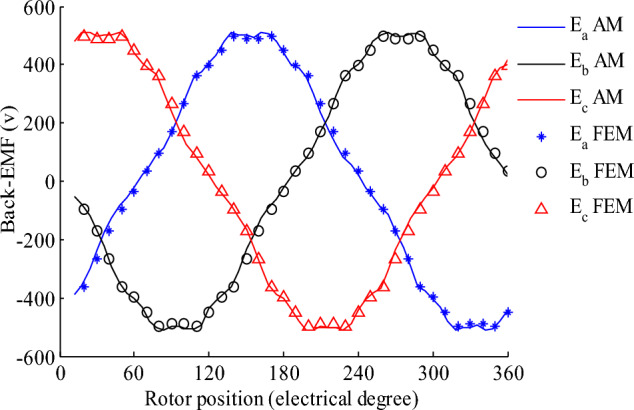
Back-EMF for all-teeth non-overlapping winding and radial magnetization.

In the following, the back-EMF is computed by considering two flux densities from two field sources, the effect of three types of the magnetization patterns is investigated in Fig. 3a–c. The back-EMF includes one cycle because the analyzed machine has one pole-pair. In the other words, electrical degree and spatial degree are same. Among the three different magnetization patterns the radial one produces superior voltage waveform. However, the curve of back-EMF is not purely sinusoidal, this type of back-EMF includes harmonics, and it increases losses^24^, so in practical applications such as HEV or aircraft the parameter of HESM such as dimensions, number of rotor and stator slots, type of stator winding, etc. should be designed properly. For example, if the dimensions of the machine are considered according to Table 2 and stator winding is assumed all-teeth, the back-EMF becomes more sinusoidal which is shown in Fig. 4.

**Table 2:** Parameters of the SHESM with the all-teeth winding.

Parameter	Values	Parameter	Values
p	2	δ_s_	0.6283 rad
Q_s_	6	β	0.4398 rad
Q_r_	4	δ_r_	0.2356 rad
R_o_	105 mm	α_p_	0.85
R_sl_	81.5 mm	B_rem_	1
R_so_	62.9 mm	μ_rm_	1.05
R_s_	57.5 mm	N, U, V_s_, V_r_	50
R_m_	56 mm	I_r_	10 A
R_r_	50 mm	I_m_	14.14 A
R_1_	26.5 mm	k_f_	0.6
L	90 mm	A_c_	1.2 mm^2^
f	50 Hz	n_s_	1500rmp

The all-teeth non-overlapping winding includes two pole-pair. Consequently, its curve consists of one/two cycles for 360 electrical/spatial degrees.

The amplitude of the Back-EMF for a HESM with specified dimensions is enlarged by increasing the rotor flux density. To increase it, the flux of the PM and the rotor winding have to be enlarged. The PM flux depends on residual magnetic flux density, so it is constant. The increasing rotor winding flux has two constraints: saturation and heat removal. Given that, the flux range of the rotor current is very small, so saturation does not occur. Table 3 represents the effective and maximum of the Back-EMF due to rotor current density. This table shows that as the rotor current density increases/decreases, the voltage value increases/decreases. Therefore, in applications where a constant terminal voltage is required, if the speed of the machine increases/decreases, the voltage can be stabilized by decreasing/increasing the rotor current density.

**Table 3:** The effective and maximum of the Back-EMF due to rotor current density.

Rotor Current Density	Effective of Back-EMF	Maximum of Back-EMF
− 10	239.5641	356.3691
− 9	246.5389	366.4273
− 8	253.5142	376.4855
− 7	260.4900	386.5436
− 6	267.4663	396.6018
− 5	274.4429	406.6600
− 4	281.4199	416.7182
− 3	288.3972	426.7764
− 2	295.3749	436.8346
− 1	302.3529	446.8927
0	309.3311	456.9509
1	316.3096	467.0091
2	323.2884	477.0673
3	330.2674	487.1255
4	337.2465	497.1837
5	344.2259	507.2418
6	351.2055	517.3000
7	358.1853	527.3582
8	365.1652	537.4164
9	372.1453	547.4746
10	379.1255	557.5328

**Figure 5:**
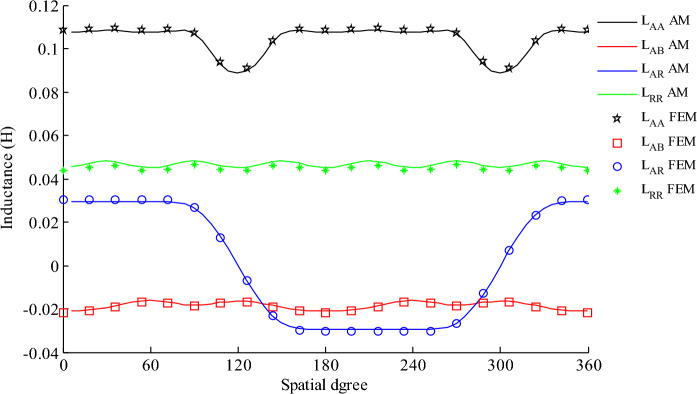
The variation of self- and mutual inductances of the stator winding with respect to spatial angle.

The self-inductance of phase A (${L}_{AA}$) and the mutual inductance between phases A and B (${L}_{AB}$) are calculated by excluding the field excitation sources and considering the reaction of the armature. After that, the self-inductance of the rotor (${L}_{RR}$) and the mutual inductance between the rotor and the stator windings (${L}_{AR}$) are determined when PM and stator winding are inactive and the rotor winding is active by Eqs. (17) and (20), respectively which are shown in Fig. 5.

The self-inductance of the rotor is almost constant because the air distance which the flux of this winding passes through doesn’t change significantly. In contrast, The stator self-inductance is not fixed. Because the rotor includes slots, when they pass in front of the stator phase, the length of the air gap increases, so the inductance decreases. The mutual inductance between the stator phases is negative because the angle between them is 120 degrees and their flux along the axis of the other phase will be proportional to cos (120). The mutual inductance between the stator and the rotor windings is positive in half and negative in the other half cycle. Because the rotor rotates and the angle between the axes of the two phases varies.

**Figure 6:**
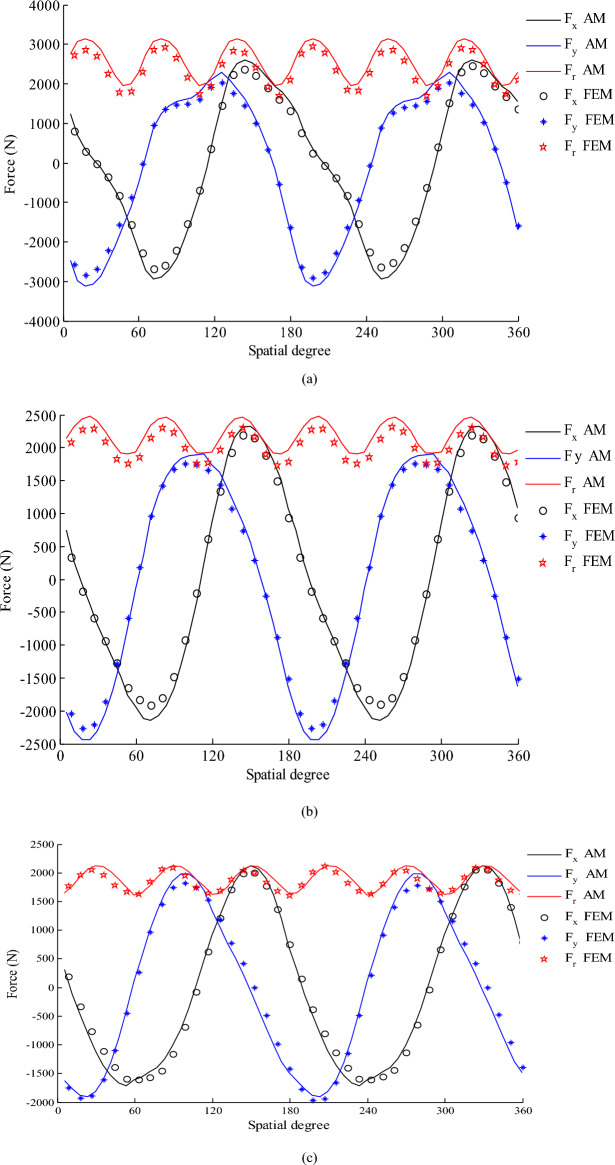
Unbalance magnetic force for alternate teeth non-overlapping winding (**a**) Radial magnetization (**b**) Parallel magnetization (**c**) Halbach magnetization.

**Figure 7:**
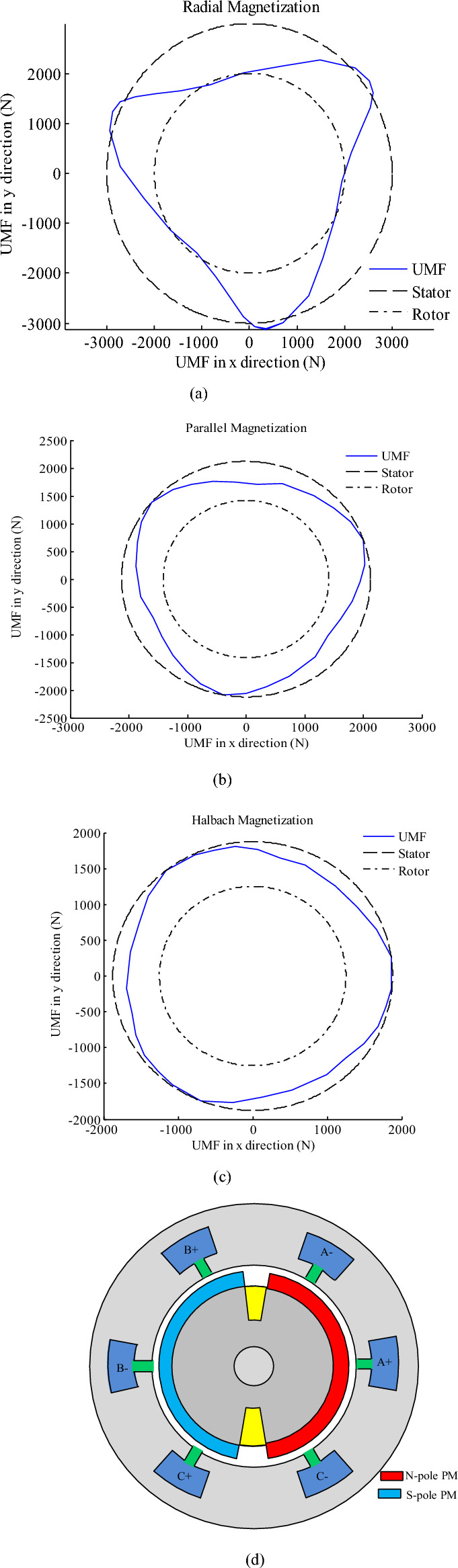
(**a**) Fy versus Fx for radial magnetization (**b**) Fy versus Fx for parallel magnetization (**c**) Fy versus Fx for Halbach magnetization (**d**) HESM topology alternate-teeth stator winding.

Finally, the comparison of the UMF estimation using 2-D analysis with those of FEM confirms the accuracy of the two-dimensional analytical method in modeling the machine and the results are presented in Fig. 6. As evident from Fig. 6, the amplitude of the unbalance magnetic force with Halbach magnetization is less than the other types. The diagram of ${F}_{y}$ in terms of ${F}_{x}$ for analyzed HESM (in Fig. 6) is drawn in Fig. 7 for three magnetization patterns. It is clear that the amplitude of UMF components have increased in the angles where stator phases locate.

The UMF is produced if there is diagonal asymmetry either in the rotor poles or in the stator core/winding. Also, UMF is created due to eccentricity. Otherwise, it must be zero. The diagonal symmetry means if a force is produced at angle θ, the HESM will also be generated at angle θ + pi. If Fig. 1 is taken into account, it doesn’t have diagonal symmetry. Therefore, the UMF will be generated.

**Figure 8:**
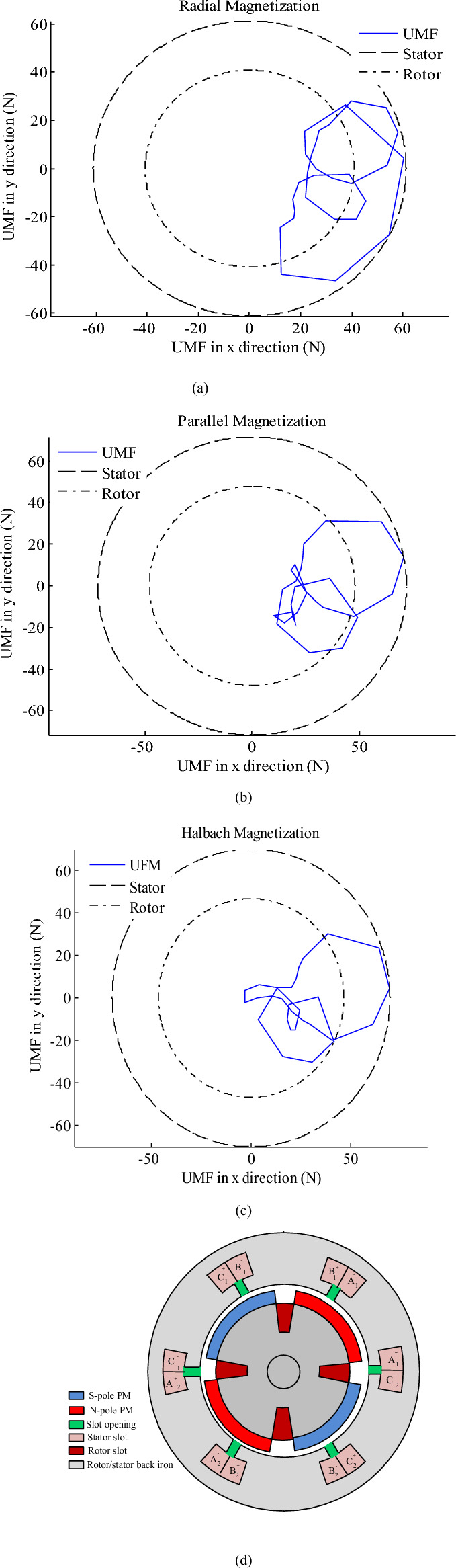
The torque components with alternate-teeth non-overlapping winding that rotor includes 2 slots and radial magnetization.

Therefore, its magnetic field is dominant and to reduce it, the field of each phase of the stator must be weakened. Therefore, winding of phases should be halved and placed in two parts of the stator. In the other words, instead of alternate-teeth winding (Fig. 7d), all-teeth winding (Fig. 8d) has been utilized. In Figs. 7 and 8, the stator and rotor are shown as two imaginary circles so that the angles where the UMF’s components are maximum can be well identified. Drawing them are not related to UMF’s components.

To further reduce the UMF, the permanent magnet and rotor winding should be modified. Therefore, the number of rotor slots increases from two to four. However, the number of poles remains constant. The new symmetrical structure for the HESM is illustrated in Fig. 8d. The reduced UMFs for last topology and radial, parallel and Halbach magnetization are presented in Fig. 8a–c. It is clear that the UMF components have been significantly reduced by proposed modifications.

**Figure 9:**
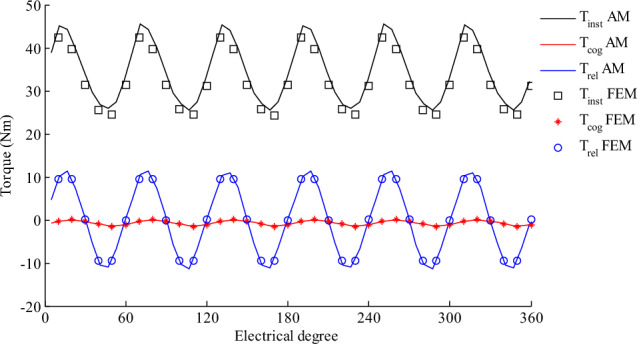
The torque components with all-teeth non-overlapping winding that rotor includes 4 slots and radial magnetization.

All torque components are calculated by (29) to (32) equations. Fig. 9 illustrates the cogging, reluctance and instantaneous torque waveforms for the HESM described in Table 1. The cogging torque is high in this structure. Yhang et al.^25^ introduced some methods to reduce torque ripple. One way to reduce the cogging torque is to use a fractional slots/pole combination. Therefore, four slots instead of two slots in the rotor is selected. Also, all-teeth winding with two pole-pairs are utilized. In improved structure (Fig. 8) not only cogging torque but also instantaneous torque fluctuation magnitude reduces. The effect of the corrections in slots/pole on the torque is shown in Fig. 9.

Another method to reduce torque ripple is changing the magnetization patterns of the PM. If radial, parallel and Halbach patterns are used, the average torque and torque ripple are obtained according to the Table 4. It is clear that Halbach magnetization pattern produces less ripple, but its average torque is less than other patterns.

**Table 4:** Average torque and torque ripple due to magnetization pattern.

Magnetization type	Average torque (Nm)	Torque ripple (Percent)
Radial	36.49	76.27
Parallel	31.49	37.75
Halbach	29.54	30.26

**Figure 10:**
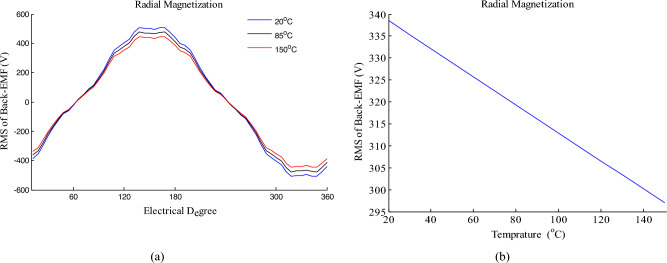
(**a**) Back-EMF curve in 20 °C, 85 °C and 150 °C (**b**) The RMS of Back-EMF based on temperature.

As shown in equation (28), the magnet residual flux density is affected by temperature. Therefore, Back-EMF and UMF change by temperature variations. Fig. 10a shows Back-EMF for HESM presented in Table 2 in 20 °C, 85 °C and 150 °C.

Also, Fig. 10b shows the RMS of Back-EMF based on temperature. It is clear that with increasing temperature, the amplitude and the RMS of Back-EMF decrease. Because of the magnet residual flux density has decreased by increasing temperature.

**Figure 11:**
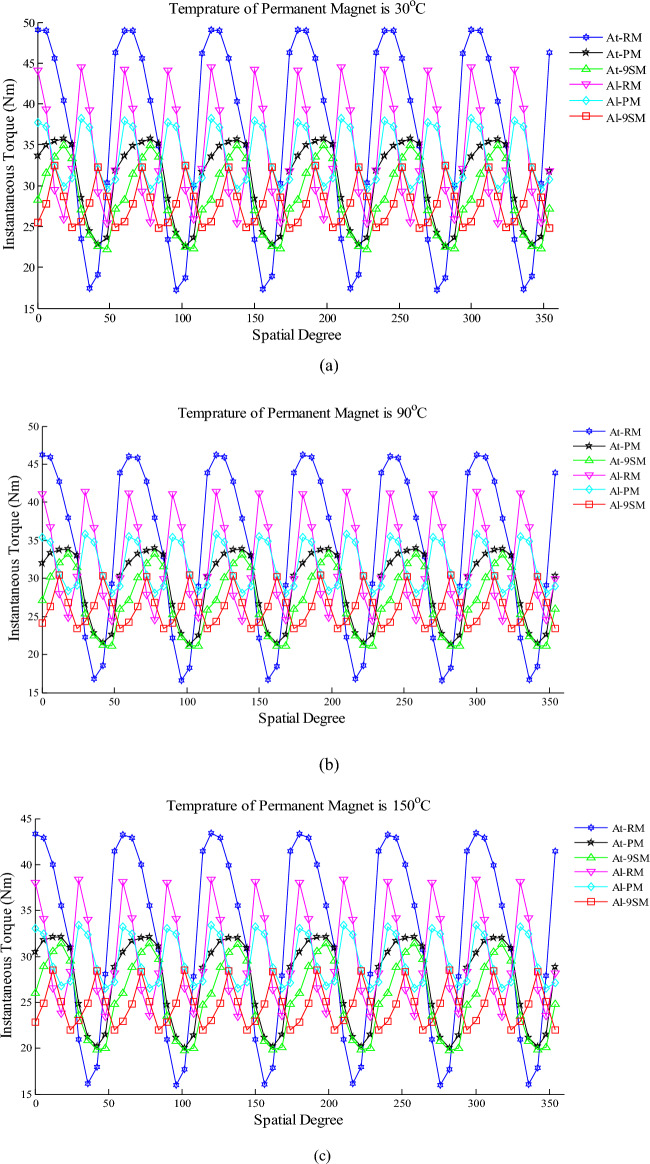
Instantaneous torque due to alternate-teeth (At) and all-teeth (Al) none overlapping armature winding and radial (RM), -parallel (PM) and 9-segment magnetization (9SM) in different temperature (**a**) 30 °C (**b**)90 °C (**c**) 150 °C.

Given that the torque is highly affected by the magnetic flux density distribution, the instantaneous torque has been computed for the radail, parallel and 9-segment magnetization patterns by alternate-teeth and all-teeth none overlapping windings in 30 °C, 90 °C and 150 °C in Fig. 11. By comparing the curves presented in this figure, it is cocluded that for the same magnetization pattern and stator winding layout, increasing the temperature leads to a decrease in torque. Because the increase in temperature reduces the magnetic field and it has a direct effect on the instantaneous torque.

### Efficiency computation

In addition to mentioned cases, the efficiency of the permanent magnet synchronous machine is computed. In this paper, it is obtained from equation (33)

33 $$\eta =\frac{{P}_{out}}{{P}_{in}}=\frac{T\omega }{{3*V}_{rms}{I}_{rms}}$$

The instantaneous torque average and the effective value of the back-EMF are illustrated in Table 5 for the HESM presented in Table 1 by different types of magnetization patterns and armature windings. Then, the efficiency has been computed based on them. By comparing the results, it is evident that the parallel magnetization pattern and all-teeth winding results in higher motor efficiency. The efficiency of machine depends on several losses such as eddy current, hysteresis, rotor and stator winding.

**Table 5:** Calculation of average torque, back-EMF and efficiency for HESMs with three different magnetization patterns and two different stator winding.

Winding	Magnetization pattern	Average torque (Nm)	RMS of Back-EMF (V)	Efficiency (%)
Alternate teeth	Radial	35.8192	501.5954	74.78
Alternate teeth	Parallel	30.8155	369.3304	87.37
Alternate teeth	Halbach	28.7029	353.3195	85.07
All-teeth	Radial	36.4907	500.5447	76.34
All-teeth	Parallel	31.4881	368.2850	89.53
All-teeth	Halbach	29.3760	352.4210	87.29

### In-wheel drive application

The efficiency of the in-wheel drive should be more than 60%^11^. The calculated efficiencies in Table 5 are in acceptable range. Moreover, the rotational speed is determined due to radius and linear speed. It can increase up to 7000 rpm^10^. The rotational speed for one/two pole-pair HESM is 3000/1500 rpm. In addition, in this application, the motor power is circa 10 kw^10^. The average torques in Tables 4 and 5 are between 28.7029 and 36.4907 Nm, so the motor power is calculated as follow.

34 $$P=T\omega =28.7029*2*3.14*50=9013 W=9.013 kW$$

35 $$P=T\omega =36.4907*2*3.14*50=11458 W=11.458 kW$$

All indicators are in reasonable range. Therefore, the proposed HESM is an appropriate option for in-wheel drive.

### Maximizing average torque

In the proposed HESM, the ratio of the magnetic flux density to the total rotor flux density has an effect on the HESM’s indicators. In this part, the goal is to maximize the average torque, so the magnetization index (MI) is defined as follow.

36 $$MI=\frac{\sqrt[2]{{{B}_{r}^{a}}_{PM}^{2}{{+B}_{\theta }^{a}}_{PM}^{2}}}{\sqrt[2]{{{B}_{r}^{a}}_{PM}^{2}{{+B}_{\theta }^{a}}_{PM}^{2}}+\sqrt[2]{{{B}_{r}^{a}}_{EC}^{2}{{+B}_{\theta }^{a}}_{EC}^{2}}}$$

where MI is magnetization index. Moreover, ${{B}_{r}^{a}}_{PM}{{ and B}_{\theta }^{a}}_{PM}$ are respectively the magnetic flux density components in the airgap due to PM in the radial and tangential directions. As well as ${{B}_{r}^{a}}_{EC}{{and B}_{\theta }^{a}}_{EC}$ are the magnetic flux density in the airgap due to EC in the radial and tangential directions, respectively.

**Figure 12:**
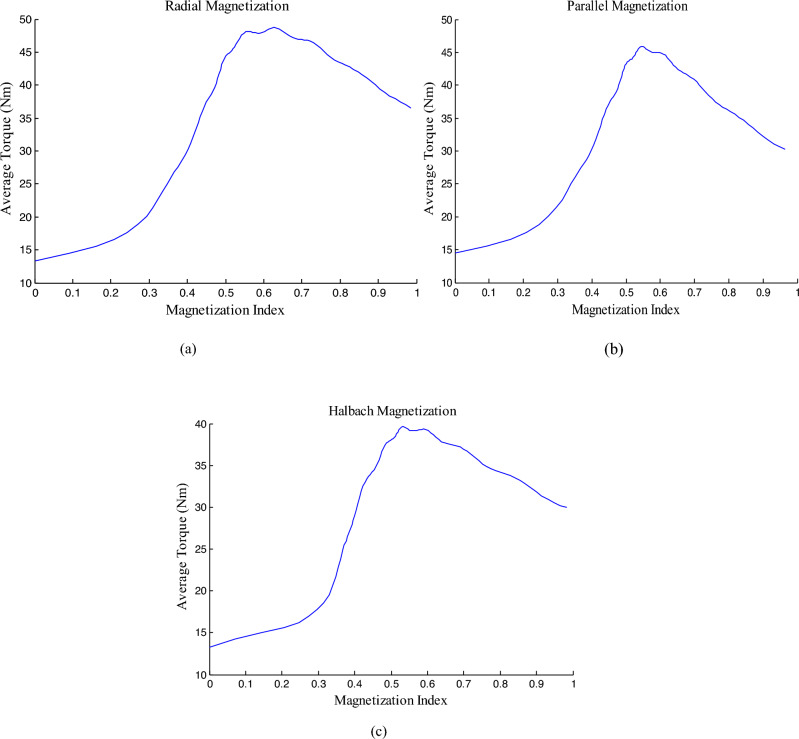
Average torque based on MI for three magnetization patterns (**a**) Radial magnetization (**b**) parallel magnetization (**c**) Halbach magnetization.

Average torque curve based on MI for three magnetization pattern types have been illustrated in Fig. 12a–c. Maximums for radial, parallel and Halbach magnetization patterns are 48.73 Nm, 45.84 Nm and 39.64 Nm, respectively. Their MIs are 0.63, 0.55 and 0.53. Therefore, to produce the maximum torque, the radial magnetization is recommended. As it is clear in all three diagrams, the complete removal of EC (MI=1) leads to a decrease in the average torque.

## Conclusion

PMSMs have been previously analyzed by 2-D method. They have constant and uncontrollable rotor flux due to PMs, so their use is limited. The proposed HESM is equipped by an auxiliary winding in the rotor, in addition to PM, so it has the merit of the rotor flux control capability. Therefore, it is a suitable option for variable speed applications such as transportation system. As well as it can stabilize the voltage despite the speed fluctuations. SHESM had been statically analyzed^21^. In this manuscript, by considering the armature reaction it has been dynamically scrutinized. At first, the radial and tangential components of the magnetic flux densities caused by PM, EC and AR are computed. Then, by considering three types of the magnetization patterns and two types of the stator winding, the Back-EMF is calculated. Besides, a suitable combination for the number of poles, slots, dimensions, type of winding and magnetization pattern were provided to generate the sinusoidal voltage. Next, the magnitude of unbalanced magnetic force is determined by three mentioned magnetization patterns. The Halbach magnetization has the lowest UMF. To further reduce the UMF to be in the acceptable range, the HESM excitation sources should be symmetrical. Therefore, the stator winding is selected as all-teeth non-overlapping winding. Also, the number of the rotor slots increases to four, but the number of the poles remains constant. After that, the effect of temperature on HESM performance indicators such as Back-EMF, UMF and instantaneous torque are investigated. As the temperature increases, not only the voltage but also the UMF and instantaneous torque decreases. Finally, the requirements of the in-wheel drive application and the MI effects on the average torque for three magnetization patterns are scrutinized. Radial magnetization is the best choice to maximize it. The good agreement between the analytical results and the FEM results confirms the accuracy of the proposed 2-D analytical model.

## Data Availability

The datasets generated and analysed during the current study are not publicly available due lab restriction but are available from the corresponding author on reasonable request.
